# Supplementary material for: Are carbon emissions trading and green financial instruments synergistic? -Comprehensive quantitative research based on content analysis
Source: PLoS One. 2024 Mar 7;19(3):e0298601. doi: 10.1371/journal.pone.0298601 (PMC10919601; doi:10.1371/journal.pone.0298601)
Supplement: S1 Data — (ZIP) [file pone.0298601.s001.zip › Data/地级市Z_F工作报告.zip!/╕ú╜¿/╧├├┼╩╨/└┤╫╘╣1⁄2╓┌║┼í╛┬φ┐╦╩2╛▌═°í┐_2003.docx]

各位代表：

　　现在，我代表市人民政府向大会作政府工作报告，请予审议，并请市政协委员和各位列席人员提出意见。

　　一、2002年和过去五年工作的回顾

　　2002年，我们在中共厦门市委的领导下，努力实践“三个代表”重要思想，积极应对加入世贸组织后的新形势，不断增强城市综合竞争力，经过全市人民的共同努力，完成了市十一届人大五次会议确定的各项任务。预计全年实现国内生产总值648亿元，增长15．5％；财政总收入126亿元，增长14．4％，其中地方级财政收入64亿元，（按可比口径）增长15．5％；城镇居民人均可支配收入增长11．6％，农民人均纯收入增长6．7％。这一年，是近年来国内生产总值、工业总产值、城镇居民收入等主要经济指标增长最快的一年。规模以上工业产值突破1000亿元，出现第一家产值超百亿元的企业，外贸进出口总额、集装箱吞吐量均占全省一半以上，境内外游客突破1000万人次，加快海湾型城市建设的战略思路基本形成，西海域整治取得实质性进展，实现厦－金个案直航，成功举办中

　　国音乐“金钟奖”和“小柴”国际音乐比赛，荣获国际“花园城市”称号。

　　一年来，我们的主要工作是：

　　着力保持工业强劲增长。实施改善工业经济运行环境政策，帮助戴尔等企业扩大生产规模；加强对新增长点的全程服务，促进PTA等项目顺利投产达产；引进外资参与橡胶厂等国有企业改制，推进卷烟厂等岛内老企业易地改造，推动传统产业优化升级；扶持厦新等重点企业依靠科技进步提高产品的市场竞争力。工业企业实现利税增长61％。

　　积极支持企业开拓市场。建立口岸通关快速联动机制，推行“大通关”，实行海港电子定舱、空港无纸化通关；推动关贸、银企协作，加大财政扶持力度，缓解出口退税严重滞后的困难；制定物流业发展规划，启动物流园区建设，增辟11条国际海、空航线；实施名牌战略，扶持企业参加境内外展销，市场进一步拓展，工业品产销率达98％。

　　不断加大固定资产投资力度。贯彻中央积极财政政策，加大对基础设施、技术改造和社会事业重点项目的投入；新引进千万美元以上外资项目33个，一批项目相继投入建设；推出医院学校、批发市场、污水处理等公用事业项目吸纳社会投资。加强重点工程调度，努力解决征地拆迁难、前期审批慢等问题，强化业主负责制和工程代建制，确保工程进度。

　　努力促进农业发展和农民增收。推动农业结构调整，加大水果水产促销，促进农民增产增收；扶持同茂、银祥等22家农林产业化龙头企业，带动农村8万多种养户；继续实施“同发展、共富裕”工程，帮扶岛外106个经济薄弱村；启动后溪流域治理，加强气象预测预报，抗御长达十个月的严重干旱；西海域基本退出养殖业，帮助养殖户陆续转产转业。

　　进一步加强城市建设与管理。编制城市发展概念规划和鼓浪屿等20多项分区规划，强化中华街区、百家村等重点部位详规，清理70多个规划调整涉及的项目，成立以专家为主体的城市规划委员会。建成市府大道、云顶隧道、海沧隧道、同安银湖大桥；推进洪山柄、新潘宅、莲坂等旧城旧村改造；试行“平改坡”，绿化岛内裸露山体，建设岛外防护林；完成北溪引水二期工程。整顿规范建筑市场秩序，严格建筑市场的准入和清出。拆除违章建筑5万多平方米。加强人民防空工程建设，举行预防和处理海域突发事件演练，颁布无居民岛屿保护和利用管理规定。

　　全面发展社会事业。开工建设厦门国家会计学院，建成东渡、前埔、金尚中学；扶持外口子弟学校，基本解决外来人员子女上学问题。完善药品集中招标采购制度，增设社区医疗卫生机构，扩大农村合作医疗覆盖范围，改善群众就医条件。加强计生工作，稳定低生育水平。改扩建影剧院、鼓浪屿音乐厅和钢琴博物馆；举办第二届群众文化艺术节；整顿网吧、录像厅等文化市场和娱乐场所；支持《厦门日报》改版，办报质量和发行量提高。广泛开展全民健身，在省运会上获金牌总数第二名。社区居委会由278个调整为165个，建成54个为老年人服务的项目。

　　切实关心群众生活。制定促进本市居民就业政策，组织提供5000多个岗位，帮扶六种重点对象再就业；支持工会开展“春雨行动”，鼓励下岗职工创业，我市就业景气指数跃升至全国第六位。完善社会保障制度，职工基本医疗保险覆盖面达83．8％，提高最低生活保障线和离退休人员生活补贴，确保失业人员和困难居民的基本生活。维护妇女、儿童的合法权益，改善残疾人的康复和教育条件。基本完成12件为民办实事项目。坚持信访联席会议制度，实行领导干部定期预约下访，解决了一批群众信访问题。推进社会治安综合治理，加强消防、交通和食品卫生专项整治，创造安全稳定的生活环境。

　　努力提高政府服务效率。基本完成政府机构改革，市政府工作部门由46个精简为41个。清理63件规章和规范性文件。巩固审批制度改革成果，改进外商投资和建设管理审批方式，进一步压缩了审批时限。推进政务公开，加强效能建设，办好政府网站。认真办理人大代表议案5件，批评、意见、建议210件，政协委员提案425件，办结率均达100％。

　　各位代表！2002年各项任务的顺利完成，为本届政府的工作画上了一个圆满句号。经过五年的努力，全市国内生产总值由359亿元增加到648亿元，年均增长14．6％；工业总产值由473亿元增加到1106亿元，年均增长21．3％；财政总收入由48亿元增加到126亿元，年均增长21．3％，其中地方级财政收入由30亿元增加到64亿元，年均增长19．8％。一些支撑经济增长、关系发展后劲、体现竞争实力的重要指标，都保持年均两位数增长。经济建设和社会发展发生了深刻的变化，集中体现在：

　　工业化层次明显提高。工业增加值占国内生产总值的51％，对经济增长的贡献率达70％。工业结构调整取得显著实效，电子、机械、化工三大产业产值接近工业总产值的70％，成为拉动全市经济增长的主导力量。形成了戴尔、柯达、厦华、厦新、厦工、厦烟、金龙、古龙等一批骨干企业和名牌产品。飞机维修、视听类电子产品、感光材料、钨制品在亚太地区占据重要地位。通士达、绿泉等一批国有老企业成功实现易地技改，提高了竞争能力。高新技术企业产值占工业总产值的比重达50．5％。企业的科研开发、市场开拓和抗风险能力增强。

　　国际化水平明显提升。五年累计合同利用外资68亿美元，实际到资超过60亿美元。全球《财富》500强企业有31家在厦投资48个项目。“投洽会”、“台交会”办会模式不断创新，全国招商引资的窗口和平台作用凸显。外贸进出口总额翻了一番，达150亿美元，地产工业品45％出口国际市场。世界前20大船务公司均在厦开展业务，厦门港与100多个港口合作，集装箱吞吐量增长3．4倍，达175万标箱，跻身全球40强。发挥港澳的桥梁作用，国际交流合作更加密切，年出入境旅客突破100万人次，国际友城增加，接待副总理级以上国宾团组23个，举办大型国际会议与展览70多场次，与90多个国家和地区进行文化教育交流合作，与海外华侨华人的联系更加紧密。

　　市场化程度明显加深。以市场为导向的各项改革深入展开，劳动力、土地、资金、技术等生产要素市场基本形成，市场在资源配置中发挥了基础性作用。住房二、三级市场初步发育，经营性土地使用权实行挂牌招标拍卖。106家市属国有企业实现改制，现代企业制度逐步形成。商贸领域进一步开放，实行进出口经营权核准登记制，引进沃尔玛等国际大型零售企业，形成了多元化竞争格局。个体私营经济在服务业和科技产业方面的作用增强。市场化改革向公用事业和社会事业领域推进。

　　城市化进程明显加速。城市化率达52％，提高了6．3个百分点。城市建成区由65平方公里扩大到102平方公里。农村工业化、城市化进程加快，3万多人口实现户籍农转非。五年共投资218亿元用于基础设施建设，建成海沧大桥、国际会展中心等一批标志性建筑，新建市政道路45公里，供水、供电、供气、污水处理能力进一步增强。完成农村电网改造，实现城乡同网同价。新建住宅1073万平方米。新建绿地1015公顷，建成区绿化覆盖率达37％。获得“全国十佳人居城市”等称号。

　　社会事业明显推进。教育优先发展的战略地位得到落实，共建厦大、集大取得成效，“两基”工作获得全国先进。率先普及高中阶段教育，高考录取率保持在70％左右。新建扩建中小学96所，社会力量办学360所。人均期望寿命等卫生指标处于国内领先水平。农村合作医疗、改水改厕取得进展。各区计划生育连年保持全省先进。爱乐乐团、小白鹭民间舞团享誉全国，一批文艺作品在全国获奖，城乡群众性文体活动活跃，公共体育设施增加，羽毛球等竞技体育取得好成绩。开展基层军民、警民共建，获得双拥模范城“四连冠”。

　　小康步伐明显加快。人民生活质量普遍提高，城镇居民人均可支配收入由8980元增加到11600元（按可比口径），农民人均纯收入由3629元增加到4720元。恩格尔系数从49．8％下降为41．2％，城乡居民储蓄存款余额增加了150亿元。平均每百户居民拥有移动电话129部、电脑46台。人均住房面积从12．7平方米提高到18．5平方米。建成经济适用房近2万套，基本解决了居民住房困难。城镇职工社会保险制度基本形成，最低生活保障线实现应保尽保，最低工资标准和失业保险金分别提高23．7％和26．6％。建立法律援助中心，完善110、120社会救助系统，人民群众对社会治安保持较高满意率。

　　以上成绩的取得，是在党中央、国务院和中共福建省委、省政府及中共厦门市委的正确领导下，在各级人大及各位代表的依法监督和有力支持下，在各级政协及各位委员的民主监督和积极参与下，全市人民齐心协力、艰苦奋斗的结果。在这里，我谨代表市人民政府，向全市人民，向各民主党派、总商会、人民团体和各界爱国人士，向离退休的老同志，向中央和省驻厦单位，向驻厦部队、武警部队和公安干警，向来厦的所有投资者和外来劳动者，向所有关心支持厦门的港澳同胞、台湾同胞、海外侨胞和国际友人，表示衷心的感谢！

　　本次大会正值政府换届，由于工作需要，部分同志将不再进入新一届政府。两任市长和这些同志在任职期间，尽心尽力，勤奋工作，为厦门经济建设和社会发展作出了重要贡献。在此，谨向他们表示衷心的感谢和敬意！

　　各位代表！五年来，我们积极应对了亚洲金融危机、台海局势变化和国内有效需求不足，抗御了台风、干旱等自然灾害，克服了特大走私案造成的恶劣影响，避免了经济发展出现大的波动，经受了严峻的考验。五年政府工作给予我们深刻的启示：必须坚持以制造业带动现代服务业，相互促进，推动结构调整和产业升级，把厦门做大做强；必须坚持外向型经济发展道路，大举“引进来”、积极“走出去”，主动参与国际分工与合作，以开放促改革促发展；必须坚持效益优先，在保持经济快速增长的同时，注重经济运行质量，着力培植财源增长点；必须坚持经济与环境的协调发展，不断营造良好的生态环境和社会环境，增强城市吸引力；必须坚持“两手都要硬”，深刻吸取特大走私案的沉痛教训，不断健全廉政机制，加强公务员队伍建设，警钟长鸣！

　　政府工作还存在一些亟待解决的问题，主要是：思想不够解放，思路不够开阔；以改革和市场经济手段解决问题的办法不多，一些深层次矛盾和问题还没有得到有效解决；部分公务员的责任心、主动性不强，办事效率跟不上。同时，还面临着一些制约经济社会发展的难题，主要是：产业链较短，开发区分散，产业布局不尽合理；规划编制的科学性和执行的严肃性不够，不能适应发展的需要；投融资体制改革滞后，公用事业项目过分依赖财政负债投入；个体私营经济发展缓慢，投资领域受到限制；岛内外发展不平衡，农村发展和农民增收困难；对台区位优势未能充分发挥等等。这些都必须在今后工作中切实加以解决。

　　二、今后五年工作的展望

　　今后五年是我市以党的十六大精神为指导，全面建设小康社会，率先基本实现现代化的关键时期。当前，我市原有的特区政策优势、区位地缘优势都在减弱，土地、劳动力使用成本上升，市场竞争、财力竞争日益加剧；同类型城市、周边城市都在抢夺发展高地，力争在新一轮竞争中抢占先机。形势逼人，不进则退。我们只有以强烈的忧患意识和高度的责任感，充分利用国内外良好的发展环境，在制度创新和扩大开放等方面再创新优势，才能继续走在全国前列。

　　中共厦门市委九届五次全体（扩大）会议，确定了加快海湾型城市建设的战略方针，这是我市贯彻党的十六大精神，全面建设小康社会的行动纲领。加快海湾型城市建设，就是通过海岛向海湾空间形态上的扩张，调整产业布局，优化经济结构，扩大经济总量，积极参与国际分工与合作，增强城市对周边区域的经济集聚、辐射和带动功能，提高厦门的城市综合竞争力。力争到2010年，基本形成现代化海湾型城市框架，把厦门建设成为生产研发基地、对台交流合作基地、航运物流中心、金融商贸中心、旅游会展中心和文化教育中心；建设成为依托闽南、面向台海、沟通国际，凸现于长江与珠江两大三角洲之间的东南沿海重要中心城市和港口风景旅游城市。

　　在加快海湾型城市建设的过程中，按照“优化岛内、拓展海湾、扩充腹地、联动发展”的总体思路，遵循“规划指导、基础先行、产业推进、城区扩展”的开发原则，做到规划上有超前意识、建设上有精品意识、开发上有经营意识、安排上有重点意识、投资上有效益意识、筹资上有民力意识，实事求是、量力而行，奋发开拓、尽力而为。

　　今后五年政府的三大任务是：

　　（一）全面构筑海湾型城市框架

　　构筑城市基础设施框架，形成便捷通畅的海陆空对外辐射通道、连接周边的区域合作通道和城市内部的快速通道，完善各开发区域的市政设施配套和信息宽带网络，使基础设施适度超前，保障开发建设和产业发展的需要。构筑新城区框架，在提升本岛城市功能的同时，推进海沧城区、马銮湾城区、集美城区、大同城区、东部城区，以及厦大－曾厝文教区、集美－杏林湾文教区、鼓浪屿艺术之岛、大嶝对台经贸旅游区的建设。构筑城市产业框架，形成岛内高层次产业区、海沧产业区、西部产业区、同集经济带、东部产业区。

　　（二）全面完成“十五”目标，制定实施“十一五”计划

　　实现四个翻番，即以2000年为基数，到2005年国内生产总值达1000亿元；工业总产值达1550亿元；财政总收入达183亿元；城镇居民人均可支配收入达2万元，农民人均纯收入达8000元。登上四个新台阶，即改革开放、城市建设、科技教育、精神文明建设上新台阶。实现四个协调发展，即三次产业之间、城市与农村、经济社会与环境、物质文明与精神文明的协调发展。在此基础上，着手制定并开始实施第十一个五年计划。

　　（三）全面提高人民生活水平

　　人民生活更加殷实，农村富余劳动力大量有序转移，城市化率达68％，城镇登记失业率控制在4％以下；城镇居民恩格尔系数降到35％，人均居住面积达20平方米；城镇居民社保覆盖面达95％以上。社会更加文明，教育、医疗条件进一步改善，万人大专以上学历人数1000人以上，人均期望寿命76．5岁，千人拥有医生6．5人、病床5．3张；政府廉洁高效，社会民主进步，“三城”建设有所推进，市民综合素质和城市文明程度明显提高。环境更加优美，人均公共绿地11平方米，工业污染源达标排放，生活垃圾无害化处理率达100％，生态环境进一步改善，人与自然更加和谐，实现经济社会可持续发展。

　　三、2003年工作的建议

　　2003年经济预期主要目标是：国内生产总值增长15％，财政总收入增长13％，其中地方级财政收入增长15％；城镇居民人均可支配收入和农民人均纯收入分别增长10％。

　　围绕政府各项工作目标，必须扎实做好十个方面工作：

　　（一）严格规划与管理，展开新一轮基础设施建设

　　提高规划水平。贯彻新的《城市规划条例》，发挥城市规划委员会作用，高标准修编涵盖岛外各区的城市总体规划。完善杏林湾、马銮湾以及海沧、杏林和集美的规划，做好鼓浪屿、黄厝、中华等片区的法定图则。调整全市土地利用总体规划，启用岛外3．6万亩新增工业用地，规划岛内14平方公里开发用地和其他储备用地。妥善处理规划的历史遗留问题。调整海域功能规划，制定生态城市建设的实施纲要。

　　加快基础设施建设。全线贯通环岛路，打通仙岳路、湖里大道。建设灌新路、集杏路、同集路延伸段、环东海域滨海旅游道路，逐步形成岛外城市交通网络。推动嵩屿港区、东通道、厦漳跨海大桥、赣龙铁路厦门段、福厦铁路、莲花水库前期工作。完成空港新货站、猴屿西航道工程。加快10万吨航道二期和海沧港区1号、5－6号泊位、9号化工码头的建设。动工兴建国际旅游客运码头。建设城市地下管理信息系统。做好石兜水库扩容，着手建设杏林湾防洪排涝工程。

　　争创“联合国人居奖”。推进莲坂片区、中华街区、百家村旧城改造和祥店、刘厝等旧村改造，推广房屋拆迁货币化安置。明确行政处罚执法权限，加大违章建筑查处力度，遏制抢种抢建行为，清除无人居住岛屿的违章设施。保护鸟类和珍稀鱼种。迁移沥青搅拌等污染源。巩固西海域整治成果，着手清理滩涂、疏浚航道、整治岸线、截流排污，做好马銮湾海堤开口的前期工作。整治湖边水库周边环境，完成同安污水处理工程建设。新建海湾公园，改建鸿山公园、金榜山公园。招标引进优良树种营造风景林，招标设计扩大夜景工程，筹办“大海·音乐”为主题的国际雕塑展，提升城市绿化美化和艺术品位。拓宽改建市区道路，提高交通管理智能化水平，落实“畅通工程”。

　　（二）进一步发展制造业，走新型工业化道路

　　做大做强支柱产业。强化对戴尔、林德、翔鹭等十家重点企业的配套，延伸电子、机械、化工等重点行业的产业链，促进厦顺铝箔、太古飞机维修、正新轮胎等扩产项目投产，推动瓶级切片等项目动工建设。编制产业规划，整合工业园区，加快海沧南部化工、灌口－东孚机械、集美北部电子、洪塘石材、西柯食品等工业园区的建设，继续推动老企业易地技改，促使产业向专业区聚集。深化名牌战略，扶持名优产品，提高市场占有率。办好跨国零售企业采购洽谈会，促进本市工业企业与跨国商业企业对接。

　　促进产业科技进步。吸引国内外科技项目来厦参与招标竞争，选择产业关联度大、市场前景和经济效益好的项目，集中优惠政策给予扶持。以数字视听、移动通讯、生物制药为重点，帮助企业提高研发能力，增强核心竞争力。支持高新技术企业设立研发中心、测试中心、中试基地，发展科技中介组织和高新技术产权交易服务机构，鼓励高等院校、科研院所在厦转化科技成果。建设通讯、集成电路、软件工程等公共技术平台。开放公共信息资源，建立全市基础性资源数据库，促进企业信息化。加快同集高新技术园区、软件园二期的建设与招商，形成新兴的产业集群。

　　（三）拓展现代服务业，提升中心城市功能

　　发展壮大现代服务业。扩大象屿保税区范围，加快东渡－象屿、航空港和海沧物流园区建设，筹建公共保税仓库。整合交通运输企业，引进国内外有实力的企业，发展第三方物流。增辟国际直达航线、国内沿海支线，兴办修船业、港口中转业，开展海陆空立体联运，拓展物流腹地。扶持连锁经营、特许经营和社区配送，促进商贸流通现代化。推动市区“农改超”，建设岛外建材、汽车等大型专业市场。改革鼓浪屿旅游管理体制，拆除法定图则以外的建筑，丰富环岛路、集美等主要景区景点。开发休闲度假、海上观光等特色旅游，建设一批五星级酒店和游乐项目。按照授权，办理五年期台湾居民来往大陆通行证，争取试办大陆居民赴金门旅游。联合周边地区发展旅游热线，争办国际旅游节，争创“中国最佳旅游城市”。扶持会展业发展，改革“投洽会”、“台交会

　　”办会方式，促进会展中心与国际会展企业合作经营。

　　主动迎接服务领域对外开放。扩大商贸服务业引资领域。根据开放时间表，做好金融、保险、证券、旅行社开放准备，抓紧寻找合作伙伴，保证届时如期引进。推动律师、会计、建筑设计、中介咨询等行业的开放。

　　（四）加强农业农村工作，促进农民收入增长

　　优化农业结构。根据“品种调优、效益调高、产业调特”的要求，健全农业科技服务体系，发展优新种苗业、无公害农业、生态观光农业和农产品加工业。加大对龙头企业扶持力度，拓宽“公司＋农户”路子，发展订单农业。建成如意果蔬、涌泉林木、浦头水产等种苗繁育中心。帮助农民畅通农产品销售渠道，提高综合效益。加强农业基础设施建设，加固澳头、后滨海堤，维修病险水库，提高抗灾能力。

　　加快农村城市化进程。按照“居住社区化、资产股份化、职业非农化、福利社保化”的思路，制定“城中村”改造方案和政策。保持农村政策延续性，完成发展用地确权，制定户籍、计生、社区建设等配套政策，结合全面建设小康社会的规划，做好黄厝、何厝、岭兜等村庄城市化试点。

　　努力增加农民收入。用政策鼓励企业吸纳农村富余劳动力，对企业新招收的本市农民工，由政府提供培训费用，提高农民就业能力。积极发展劳务输出。落实好西海域养殖户转产转业措施。继续实施“同发展、共富裕”工程，加大对老区、边远村庄扶持力度，重点帮助32个经济薄弱村。缓征农业税和农业特产税，进一步减轻农民负担。

　　（五）推动对外经贸合作，凸显开放前沿地位

　　提高引资水平。实行招商项目各级领导跟踪服务制、外资项目前期工作代办承包制，成立重大项目专门服务小组，促进项目签约和履约。制定延长产业链的配套招商措施，研究吸引国内外企业来厦设立研发机构、销售总部的办法。服务好现有外资企业，及时帮助解决问题，促其增资扩产。加强与国际投资促进机构的合作，重视投资环境整体形象的推介，加大招商项目储备。改进招商引资考评，落实奖励措施，推广委托招商和代理招商。强化企业、商会和行业协会的招商功能。

　　优化口岸环境。适应制造业的快速发展，进一步提高通关效率。协助口岸单位开展联合查验、直通报检和信誉通关，落实提前报关报检、实货放行的措施。建立口岸综合信息平台，推动重点企业与海关、检验检疫联网，扩大“无纸化通关”试点范围。规范口岸中介服务，进一步降低收费，增强厦门口岸的吸引力。

　　扩大对外贸易。加强对世贸组织规则的研究利用，及时收集出口商品、出口市场、非关税贸易壁垒的信息，发挥反倾销应诉服务中心作用，协助有关企业做好产业损害调查。扩大出口退税账户托管贷款业务，缓解外贸企业资金周转困难。降低外贸经营权准入门槛，允许外商投资经营进出口贸易。尽早研究我国与东盟十国结成自由贸易区带来的影响，引导企业开辟新兴市场。调整商品结构，增加科技含量，提高出口效益。重视和加强进口工作，促进商贸业发展。支持有条件的企业“走出去”，在海外投资设厂，发展境外加工贸易。加强厦－台经贸合作，扩大对金门的货物贸易和人员往来，促进两岸直接“三通”。

　　加强区域经济协作。大力引进内资，拓展内销市场。密切与闽、粤、赣十三地市以及闽西南五地市的合作，重点促进产业衔接配套。继续做好对西藏、宁夏、重庆、新疆有关地区的对口帮扶，推进企业间合作。

　　（六）加快体制改革，推进制度创新

　　深化投融资体制改革。凡一般经营性项目都可以向社会资本开放。加大基础设施和社会事业的开放力度，逐渐减少财政投入，鼓励外资和民间资本进入，政策上一视同仁。选择部分政府投资建成的交通、供水、批发市场等经营性基础设施，向国内外投资者出让股权、经营权，将回收资金用于滚动开发新的建设项目。

　　加大国有企业改革力度。整合五家国投公司、六家授权经营集团，撤销一批管理型集团公司，减少管理层次，完善国有资产管理体制。加快市属企业的改制，国有资本从一般性竞争领域有序退出。通过企业制度创新、主辅分离，加快厦工、港务等企业集团的专业化改造，形成具有经济规模和竞争能力的优势企业。推动集体企业改制，鼓励发展多种形式的股份合作经济。

　　大力改革公用事业。促进公用事业股权多元化、经营市场化、管理企业化。改变自来水、污水处理等企业股权结构，推出城市绿地、垃圾处理、海域保洁等项目向社会公开招标经营。

　　推进事业单位改革。按照政事分开和市场化原则，基本完成人员聘用制，实现由身份管理向岗位管理转变，推进收入分配制度改革。生产经营类事业单位要逐步改制为企业。进一步下放中小学管理权限，完善竞争激励机制，实行校长职级制。市属综合医院实行全员聘任，其他有条件的医院陆续改制，办成股份制医院或民营医院。

　　（七）做好财税金融工作，促进经济发展

　　集约使用财政资金。加大对城市规划、科技进步、扩大就业、社会保障等方面的投入。发挥财税的经济杠杆作用，培育地方新财源。合理配置财力资源，统筹用好预算内外资金，调动和引导社会资金，提高财政资金的引导作用和使用效率。推进财政国库管理制度改革，做好部门会计集中核算试点，组建财政国库支付中心，建立国库单一账户体系，构建公共财政基本框架。健全政府偿债机制，防范财政风险。

　　培育发展金融市场。积极引进国外金融保险机构和国内股份制银行。加快改革地方金融机构，吸引国内外资本参股市商业银行。推动银行开展应收账款贷款、国际保理等业务，拓展融资新渠道。充分利用厦门金融存量资金。惩处逃废银行债务行为，创建厦门金融安全区。扶持上市公司通过重组增发新股、配股，推动环境科技等企业上市。

　　（八）充分调动积极因素，创造良好发展环境

　　赋予各区更大的管理自主权。着手研究区划调整，明确各行政区和海沧开发区的功能定位和产业方向。完善市、区财政管理体制，合理划分事权财权，制定跨区开发税收分享政策。进一步简政放权，除规划、土地、公共安全和环保外，其他权力下放到区。修改人才准入条件，适度放宽岛外各区的户籍政策。按照“一级规划、二级建设、三级管理”原则，理顺建设管理体制，增强区级经济竞争力。

　　继续改善投资环境。落实减轻企业负担各项规定，清理行政事业收费，降低企业经营成本。营造重商、亲商的社会氛围，定期举行政府与外资、台资、个体私营企业，与总商会、青年企业家协会的恳谈会，及时听取意见，共商发展对策。组织贯彻实施《中小企业促进法》，建立中小企业发展基金，发挥中小企业担保公司作用，制定对个体私营经济的扶持政策，创造平等竞争的市场环境。鼓励发展经济类行业协会和中介组织。整顿施工环境，依法处理强卖强包、阻工阻路的行为。

　　进一步开发人力资源。加强对大中专院校毕业生和用人单位的政策指导。引进更多高层次人才和实用技术型人才，实行人才居住证制度，给予户籍市民待遇。根据企业规模和财税贡献，确定个体私营企业人才落户指标。建立人才创业基金，吸引更多的海外留学人员来厦创业。完善职业分类工资指导线制度。营造尊重人才、鼓励创新，善待挫折、宽容失败的创业氛围。

　　继续整顿和规范市场经济秩序。健全社会信用体系，加强企业信用评级，加快建设社会信用信息系统。继续严厉打击走私贩私、偷逃骗税、侵犯知识产权等行为。健全市场准入和退出机制。

　　维护社会安定稳定。明确安全生产责任，果断消除安全隐患，依法查处事故责任人，避免发生重特大事故。加强社会治安综合治理，深化社区警务建设，组建协警员队伍，搞好群防群治。打击“六合彩”，查禁“黄赌毒”，对危害群众生命财产安全的严重犯罪，露头就打。加强国防教育，提高国家安全意识，防范和打击境内外敌对势力的渗透破坏活动。组织民防演练，加强海防管理。继续做好新形势下的双拥工作，关心复转军人，支持驻厦部队、武警部队、民兵预备役部队建设，共创全国双拥模范城“五连冠”。

　　（九）加强精神文明建设，繁荣各项社会事业

　　优化教育资源，合理调整学校布局，扩建改建现有校舍，确保明年新增2000多名初中生按期就学。推进岛内中等职业学校、外国语学校高中部迁建岛外。鼓励社会力量办学，在政策优惠等方面与公办学校一视同仁。扶持和规范民办教育、幼儿教育，不断提高教学质量。继续共建厦大、集大，支持重点院系建设。加快国家会计学院以及电大、职大、教育学院合并后新校的建设，做好厦门理工学院、医高专、华侨大学厦门校区的前期工作。办好集美学村90周年庆典。建设市图书馆新馆和陈嘉庚纪念馆，推进厦门书城、博物馆前期工作。支持电影院线改革、文艺团体改制，发展文化产业。鼓励创作反映特区创业历程和精神风貌的文艺精品。完善医疗保险制度，扩大医保IC卡定点医院范围。推进药品流通体制改革，全面实行药品招标采购。加强预防保健、妇幼保健、老年保

　　健工作，加快建设第一医院和中山医院新楼、同安医院、中医院新院和妇幼保健院。增加社区卫生服务机构布点，提升农村医疗卫生服务水平，扩大农村合作医疗覆盖面。促进优生优育，提高人口素质。增加体育设施，改造体育中心，建设嘉庚体育馆、同安体育馆。深化报业、广电体制改革，组建报业、广电集团，加快有线电视网络数字化改造。推进青少年活动阵地建设。加强科普教育。鼓励发展民办社会福利事业。做好侨务、民族与宗教工作。深入开展基层群众性精神文明创建活动，继续贯彻《公民道德建设实施纲要》，倡导敬老爱幼、帮困助残的传统美德，营造健康和谐、积极向上的社会氛围。

　　（十）维护群众根本利益，提高人民生活水平

　　关心群众生活。做好新一批为民办实事项目。加强社区公共服务设施建设，促进社区内文化体育资源共享，改善生活环境，方便群众生活。继续建设提供一批经济适用房和廉租房，改善特困群众住房条件。帮助困难群众解决子女入学问题，保证义务教育阶段所有适龄少年都能入学。大力治理餐桌污染，积极推广放心食品，强化农产品检验检疫。健全劳动力市场服务体系，建设覆盖社区的就业服务信息网。落实再就业扶持政策，加强就业技能培训。进一步拓宽再就业渠道，大力发展社区服务业，推广临时就业、劳务派遣和钟点工等灵活就业方式。加强劳动监察，维护外来劳动者的合法权益，维持劳动关系的和谐稳定。主动征集人民建议，高度重视群众信访，经常身临实地贴近群众，动手解决热点难点问题。

　　继续完善社会保障制度。把职工生育保险纳入社会保险，统一社保经办机构。加强征缴、清理拖欠，确保社保基金安全运营，确保失业保险金、养老金按时足额发放、及时接续失业人员的社会保险，完善城乡居民最低生活保障制度。健全街镇劳动保障服务站和社区服务点，把失业人员、退休人员管理，与就业服务、社会保险工作结合起来，实现“一站式”服务。

　　四、建设对人民高度负责的政府

　　人民政府要身体力行“三个代表”重要思想，把实现、发展和维护好人民群众的利益，作为一切工作的出发点和落脚点，本着对全市200多万人民高度负责的精神，聚精会神搞建设，殚精竭虑谋发展，使我们的政府成为人民满意的政府。

　　（一）增强发展观念，奋力开拓进取

　　焕发朝气，敢于创新。特区发展的实践证明，每一轮的大发展，都是以思想解放为先导，以观念突破为开端。我们要从不合时宜的思想观念、做法规定和管理体制的束缚中解放出来，经常反思自己的思维方式、行政方式、工作方式，经常检查制定的政策、采取的措施、开展的工作，是不是做到了与时俱进，有没有坚定地突出第一要务，能不能充分地调动积极因素。只要对发展有利，对群众有利，对全局有利，就要敢试敢闯。吸收借鉴国内外发展的经验教训，追踪研究上海、深圳推进发展的最新动态，善于用改革的思路、发展的办法、市场经济的手段，不断解决深层次的矛盾和问题，再创特区发展的旺盛活力。

　　弘扬正气，敢于负责。发展是人民最高利益，是党和政府最大凝聚力。我们要以厦门新一轮发展为根本职责，强化政府责任、部门责任、领导者责任，围绕增长目标、实际任务、具体项目落实责任，建立健全责任考评机制和责任追究机制，把敢负责、抓落实作为政治品质的集中体现，把求实效、创业绩作为能力才干的鉴别标准。激励全市公务员在各自岗位上，紧扣发展主题，尽心尽力尽责。

　　增强锐气，敢于突破。破解发展中的难点问题，事关发展大局，破解一个难题就能赢得一步发展，破解一系列难题就能赢得大的发展。要直面矛盾、敢于碰硬，不回避、不推诿、不拖延。破解难题，要听取各种意见，制定周密方案，做过细的思想工作，尽可能兼顾各方利益。当利益产生冲突时，必须以发展大局为重，只有发展了，各方才会受益。要突破阻碍生产力发展的体制性障碍，突破影响特区发展的人为阻力，盯紧抓住当前征地拆迁、旧村（城）改造、规划调整、环境治理、企事业单位改革、个体私营经济发展等难题，统一认识，形成合力，一个一个地研究探索，一个一个地突破解决，把我们的事业一步一步地向前推进。

　　（二）增强法制观念，坚持依法行政

　　自觉坚持向人大及其常委会报告工作的制度，严格执行人大及其常委会的各项决议，主动接受人大及其常委会的监督，认真办理人大代表的议案和建议、批评、意见。密切配合人大，搞好法规草案的拟定工作。改进和完善政府制定规章的程序，增强工作透明度。加强对公务员尤其是各级领导干部的法制教育，强化法律意识，切实依法行政。理顺行政执法体制，规范行政执法主体，全面推行行政执法责任制、执法公示制，文明公正执法，提高行政机关执法水平。强化执法监督，做好行政复议和行政诉讼应诉工作。加强基层政权民主建设，做好村（居）组织换届选举。坚持求真务实，优化统计环境，坚决不搞虚报浮夸，不搞脱离群众的“形象工程”。提高公信力，建设“诚信政府”。

　　（三）增强民主观念，实行透明行政

　　尊重和维护人民政协的职责，完善与政协的联系和协商制度，认真办理政协提案，主动接受民主监督。对牵涉面广、群众关注的重大事项，决策之前充分征求各民主党派、总商会、无党派人士和社会各界的意见。建立健全规划、价格等听证会制度，保证决策符合最广大人民的根本利益和普遍意愿。进一步推进政务公开，政府工作除属于国家机密和规定保密的事项外，一律向社会公开。尝试开放市政府常务会，各部门也要逐步实现决策透明化，保障人民群众的知情权、参与权和监督权。发挥工会、共青团、妇联和其他群众组织的作用，拓宽社情民意的反映渠道，主动接受新闻媒体的舆论监督，促进政府决策的民主化和科学化。

　　（四）增强效能观念，提高行政效率

　　参照国际惯例和先进城市做法，深化行政审批制度改革，避免重复审批、交叉审批，最大限度削减审批项目。筹建政务服务中心，加快电子政务建设，推广网上办公，推行网上并联审批，进一步简化手续、缩短时限。落实服务承诺制、首问负责制等十项制度。欢迎人大代表、政协委员和政府服务对象，对政府及各部门开展评议活动。加强政务督查，做到令行禁止，确保政令畅通。加强行政监察，完善投诉机制，加大投诉处理力度，落实行政过错追究制。严格出国审批制度，限制空泛招商和一般性考察。严格控制检查评比，减少庆典、表彰等事务性活动，下大力压缩会议，减少文件，开会、讲话、发文要解决问题。

　　（五）增强自律观念，加强廉政建设

　　要严格执行廉洁从政各项规定，做到任何时候都不以权谋私。进一步搞好廉洁自律、查处大案要案、纠正部门和行业不正之风的工作，始终保持遏制腐败的强劲势头。领导干部要管好自己的配偶、子女和身边工作人员，在反腐倡廉中起表率作用。对一般违规问题，要早发现、早批评、早纠正。提倡勤俭办事业，反对铺张浪费。规范“用权、理财、管人”行为，加强对财政预算资金使用的审计监督，严格“收支两条线”管理，严格实行领导干部经济责任审计。公开政府采购、土地批租、工程招标、产权交易等容易滋生腐败的环节，主动接受各方监督检查，从源头上防止腐败。谁敢冒犯，决不姑息。

　　（六）增强素质观念，加强队伍建设

　　深入推进机构改革和人事制度改革，扩大竞争上岗和轮岗范围，推动干部交流。鼓励公务员参加继续教育，选送有潜质的公务员到国内外高校接受培训，提高业务素质和行政水平。优化公务员队伍结构，选拔录用更多的社会优秀人才进入公务员队伍，尝试政府雇员制。坚持德才兼备、实绩突出、群众公认的用人导向，不断激发工作热情，使广大公务员尤其是各级领导干部积极适应新一轮发展的要求，努力具备经济头脑、国际眼光、服务意识和法律素养，深怀爱民之心、善谋富民之策、恪守为民之责、多办利民之事，为厦门发展建功立业。

　　各位代表！让我们高举邓小平理论伟大旗帜，全面贯彻“三个代表”重要思想，在以胡锦涛同志为总书记的党中央的领导下，按照中共厦门市委确定的发展战略，脚踏实地，勤奋工作，确保完成经济社会发展的各项任务，为创造厦门人民的美好生活而努力奋斗！
